# Implementation project of the non-pneumatic anti-shock garment and m-communication to enhance maternal health care in rural Tanzania

**DOI:** 10.1186/s12978-018-0613-5

**Published:** 2018-10-19

**Authors:** Godfrey Mbaruku, Michelle Skaer Therrien, Robert Tillya, Selemani Mbuyita, Zacharia Mtema, Iddajovana Kinyonge, Ritha Godfrey, Silas Temu, Suellen Miller

**Affiliations:** 1 Ifakara Health Institute, Dar es Salaam, Tanzania; 2 University of California, San Francisco, School of Medicine, Department of Obstetrics, Gynecology, and Reproductive Sciences, Safe Motherhood Program, California, USA

## Abstract

**Background:**

Obstetric hemorrhage (OH) remains one of the leading causes of maternal mortality, particularly in rural Africa. Tanzania has a high maternal mortality ratio, and approximately 80% of the population accesses health care lower level facilities, unable to provide Comprehensive Emergency Obstetric Care (CEmOC). The non-pneumatic anti-shock garment (NASG) has been demonstrated to reduce mortality as it buys time for women in shock to be transported to or to overcome delays at referral facilities.

**Methods:**

This report describes one component of an ongoing maternal health improvement project, Empower, implemented in 280 facilities in four regions in rural Tanzania. The NASG along with a Closed User Group (CUG) mobile phone network were implemented within the overall EmOC project. Simulation trainings, repeated trainings, and close hands-on supportive supervision via site visits and via the CUG network were the training/learning methods. Data collection was conducted via the CUG network, with a limited data collection form, which also included free text options for project improvement. One-to-one interviews were also conducted. Outcome Indicators included appropriate use of NASG for women with hypovolemic shock We also compared baseline case fatality rates (CFR) from OH with endline CFRs.

Data were analyzed using cohort study Risk Ratio (RR). Qualitative data analysis was conducted by content analysis.

**Results:**

Of the 1713 women with OH, 419 (24.5%) met project hypovolemic shock criteria, the NASG was applied to 70.8% (*n* = 297), indicating high acceptability and utilization. CFR at baseline (1.70) compared to CFR at endline (0.76) showed a temporal association of a 67% reduced risk for women during the project period (RR: 0.33, 95% CI = .19, .60). Qualitative feedback was used to make course corrections during the project to enhance training and implementation.

**Conclusions:**

This implementation project with 280 facilities and over 1000 providers supported via CUG demonstrated that NASG can have high uptake and appropriate use for hypovolemic shock secondary to OH. With the proper implementation strategies, NASG utilization can be high and should be associated with decreased mortality among mothers at risk of death from obstetric hemorrhage.

## Plain English summary

Obstetric hemorrhage (OH), defined as excessive bleeding during pregnancy, childbirth, or the first 24 h after childbirth, remains a leading cause of death for women, particularly in rural Africa. OH can lead to shock, a state in which the body’s vital organs lack enough oxygen, which leads to organ failure and death. In Tanzania women die from bleeding and shock due to long distances from emergency health services, Comprehensive Emergency Obstetric Care (CEmOC). One way this problem has been addressed is with the use of a lightweight, cost-efficient compression garment, the non-pneumatic anti-shock garment (NASG), which stabilizes women who have lost excessive blood, allowing them to survive delays in travelling to or receiving care at CEmOC facilities. Until now, there have been questions if NASGs can be implemented effectively in rural areas.

This study introduced the NASG at 280 health facilities in rural Tanzania along with a Closed User Group (CUG) mobile phone network. A CUG network is a service from a cellular phone company which allows phone calls to be made at no charge to other phones on the same network.

Data was collected on 1713 women with OH, and 24.5% (419) of these women had signs of shock. The NASG was applied to ~ 71%% (*n* = 297) of women with shock, showing high uptake and appropriate use. The rate of women dying from OH during the study at facilities, called the case fatality rate (CFR), was reduced during the study period.

## Background

Obstetric hemorrhage (OH) is one of the leading causes of maternal deaths, accounting for 33.9% of maternal deaths in Africa to 13.4% of maternal deaths in developed countries, with the majority of deaths occurring in low resource settings (LRCs) [1, 2]. Deaths due to hemorrhage are highly preventable if managed appropriately, but deaths frequently occur in rural areas far from comprehensive emergency obstetric care (CEmOC) centers able to provide surgery and blood transfusions, the definitive treatments for severe hemorrhage with hypovolemic shock [3, 4]. One first aid management tool for hypovolemic shock secondary to obstetric hemorrhage is the non-pneumatic anti-shock garment (NASG). This low-technology, easy to apply, circumferential pressure device, is made of stretchy compression neoprene and closes tightly with Velcro, reversing shock, restoring vital signs, and decreasing blood loss in the pelvis and uterus [5]. This first aid device buys valuable time, to enable transfer from rural and lower level facilities, and during the often-long delays for blood transfusions or surgeries that can occur, even in tertiary and university teaching hospitals in LRCs.

Morbidity and mortality outcomes using the NASG have been positively compared to outcomes with standard treatment of shock/hemorrhage in women with a variety of OH etiologies, and the NASG was found to be effective in decreasing blood loss by over 50% [6–9]. A systematic review including 5 studies and 1247 women [10] with OH /shock found a decreased mortality of 48% (Relative Risk (RR) 0.52 (95% Confidence interval (CI) 0.36 to 0.77) at referral, tertiary-level facilities. A Cluster Randomized Clinical Trial (CRCT) was conducted in Zimbabwe and Zambia [9] to determine if early application of the NASG at the primary health facility level improved outcomes compared to later application at the referral facilities. The reduction in mortality was clinically significant at 55%, but there was an inadequate sample of women in hypovolemic shock (actual sample size, *n* = 880 vs. the predicted *n* = 2400) necessary to have adequate power to determine statistical significance [11].

The NASG is currently recommended by the International Federation of Gynecologists and Obstetricians (FIGO) [12] and the World Health Organization (WHO) [13] and can be found in PPH guidelines and manuals, such as WHO (Managing Complications 2nd edition) [14], Global Library of Women’s Medicine (GLOWM) PPH Recommendations [15], and JHPIEGO’s Helping Mothers Survive Bleeding After Birth [16], as well as in JHPIEGO’s 2018, 5-year report “Survive and Thrive” [17]. A healthcare technology assessment performed for WHO [18] resulted in a positive recommendation for including the NASG in Emergency Maternal and Obstetric Care (EMOC) management. In 2015 the UN Commission on Life-Saving Commodities for Women and Children (UNCoLSC), the Clinton Health Access Initiative, Inc. (CHAI), the Safe Motherhood Program at the University of California, San Francisco (UCSF), and the Blue Fuzion Group entered into an agreement to decrease costs of the NASG by 75% in order to enhance NASG scale up in LMICs [19].

Despite these global recommendations and endorsements and despite pilot implementation and scale up projects conducted in India [20, 21], Nigeria [22], Ethiopia [23, 24], Niger [25], Timor Leste [26], and Colombia [27], there have been fewer publications on how the NASG is accepted and used by clinicians and health systems [26, 28–31].

The publications/reports that have measured utilization reported a range from 14% to 47% in Nigeria and Timor Leste [26, 28, 30]. While not published, a few presentations on a two-phase evaluation of a national PPH project in Niger, which introduced misoprostol, uterine balloon tamponade (UBT), and NASG as a PPH prevention and treatment package, reported extremely low rates of use and raised the issue of a need for strategic planning around the use of the new technologies, NASG (and UBT) [32–34].

This paper will focus on utilization rates, provider acceptability, barriers to utilization, and those elements of training and supportive supervision in-person and via use of a Closed User Group (CUG) phone system, which fostered higher, appropriate rates of NASG use in a large maternal health implementation project in rural Tanzania.

## Methods

The 2010 Demographic and Health Survey revealed that in 5 years, Tanzania reduced maternal mortality from 578 to 454 maternal deaths per 100,000 live births [35, 36]. While emergency obstetric care (EmOC) is available at regional hospitals, a recent study of 22,243 live births in rural districts of Tanzania found that only 29% of deliveries occurred in a hospital [3].

This project was a component of the Empower II Project conducted by the Ifakara Health Institute (IHI), which implements Maternal, Newborn and Child Health (MNCH) interventions in real world settings to produce learning for improvements and scale up. Empower I lasted from July 2007 to June 2012 and Empower II launched in March 2013. Collectively, Empower works in four rural regions of Tanzania and targets a total population of 3,919,342 of which 813,092 are women of reproductive age. The NASG/CUG component described below was conducted from November 2014 to June 2016. Members of the University of California, San Francisco (UCSF) Safe Motherhood Program served as consultants for the NASG portion of the project.

**Fig. 1:**
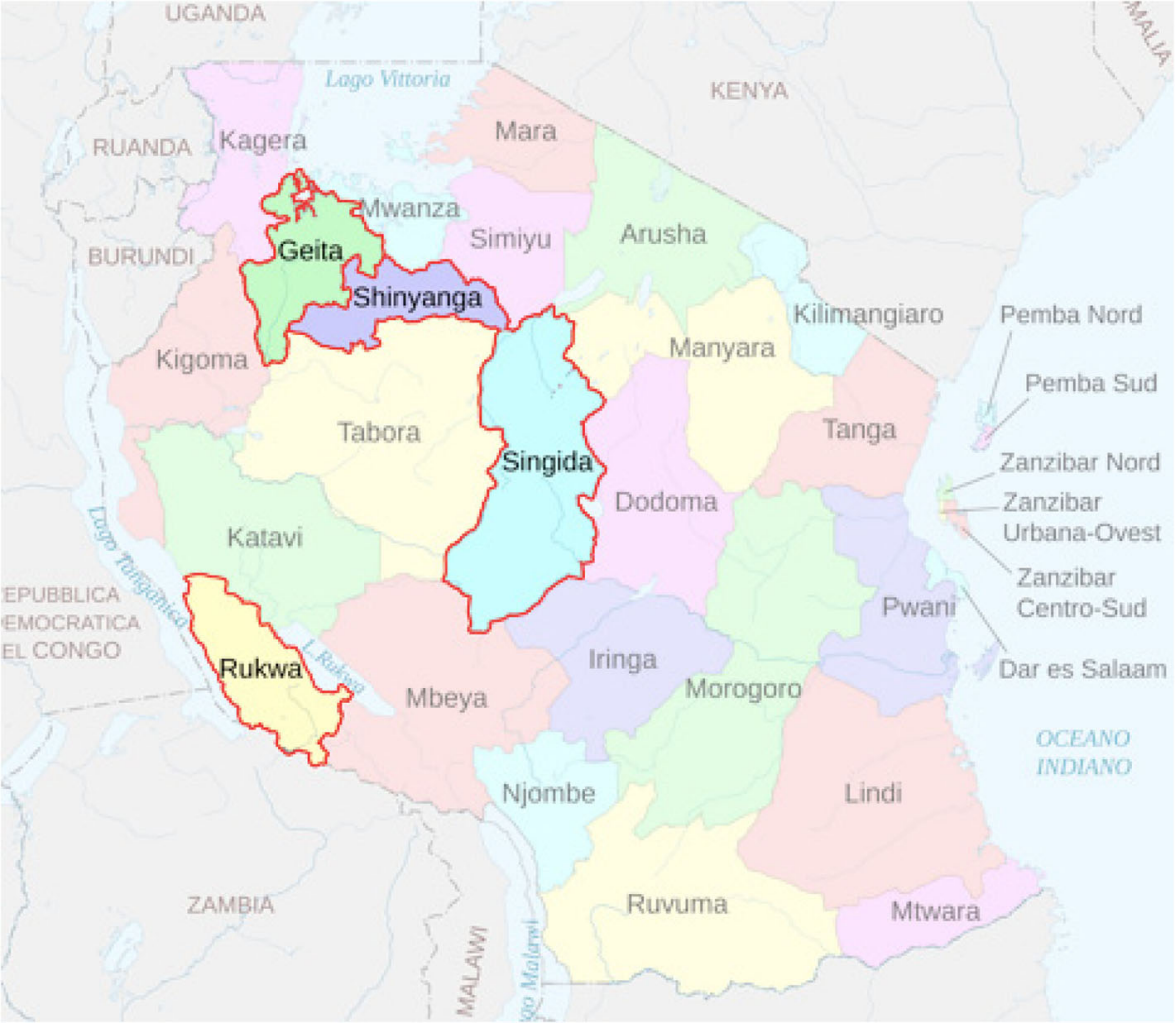
Intervention regions. Study conducted in 2 districts in each of the 4 regions

This project component addressed challenges around prompt management of OH and around OH referral between facilities through the introduction of the NASG (LifeWrap-NASG, Hong Kong) along with a Closed User Group (CUG) mobile phone network for public dispensaries, health centers, and three public referral hospitals, as well as three private referral hospitals in the eight districts of Geita, Nyang’hwale, Kalambo, Sumbawanga, Singida, Ikungi, Shinyanga, and Kahama (Fig. 1 for a map of the regions). A total of 280 facilities were enrolled: 17 Comprehensive Emergency Obstetric Care (CEmOC) facilities and 263 Basic Emergency Obstetric Care (BEmOC) facilities (17 health centers and 246 dispensaries). To qualify as a CEmOC referral facility, a site needed to have an operating theater and the capacity to perform a blood transfusion. BEmOC level health centers served a population of 50,000 people and provide inpatient care. Dispensaries provided the lowest level of care, serving a population of 10,000 people, and offering delivery beds and outpatient services. While dispensaries were intended to provide BEmOC care, not every dispensary was completely able to do so (the majority were not able to perform these signal functions: parenteral anticonvulsants, removal of retained product, and assisted instrumental delivery).

In addition to the NASG, the project introduced mobile phones on a CUG network. Five hundred eight cell phones were supplied, three per CEmOC facility, 2 per BEmOC facility, and four for each of the 8 Council Health Management Teams (CHMT) with phone service from the Airtel telecommunications company. Phones on a CUG network may call other phones within the network without airtime fees, so that health workers could communicate about patients, call for an ambulance, and contact IHI project staff for information and support.

The study trained participants to use the NASG for pregnant and postpartum women with hypovolemic shock secondary to obstetric hemorrhage of any etiology: ectopic pregnancy, molar pregnancy, complications of abortion, retained placenta, placenta previa, uterine atony, uterine rupture, and genital trauma. Women were classified as having hypovolemic shock if they had any amount of bleeding combined with either a systolic blood pressure of ≤80 mmHg or altered consciousness. Women were included whether the hemorrhage began at home, at the facility, or at another site.

### Training

Three phases of training were conducted: a Training of Trainers (TOT) in October 2014, a series of Phase 1 trainings for CEmOC facilities was conducted in November 2014, and a series of Phase 2 training for BEmOC facilities was conducted in February 2015. Sessions specific to the NASG were 1.5–2 days in length and taught a curriculum developed by the UCSF Safe Motherhood Program [curriculum and training videos in English and a video of training in Swahili are available at: http://www.safemotherhood.ucsf.edu/resources/videos/, the NASG/CUG content was incorporated into scheduled Empower Emergency Obstetric Care (EmOC) trainings.

At the TOT 19 trainers were certified to support ongoing cascade trainings as well as project supervision. Only trainers who were able to pass a skills test and had a written exam score of > 70% were certified to train for the project. Phase 1 consisted of four trainings, one per region at CEmOC facilities. Each facility sent four to five CEmOC providers, who were trained in clinical management of women with hypovolemic shock with the NASG and how to train other facility staff in NASG use. A phased approach was used, beginning project implementation only at district hospitals and other CEmOC health facilities for the first 3 months of the program (December 2014 – February 2015), before moving to Phase 2 training and introduction of NASGs to lower level facilities that referred patients to these CEmOC facilities. Phase 2 consisted of 16 trainings in Swahili for BEmOC health center and dispensaries, four trainings per region. The trainings were conducted in March–April 2015, BEmOC facilities sent two staff members to represent each dispensary and 3–4 staff for each health center. These trainings were integrated with another scheduled Empower training on maternal health service provision. A total of 863 staff were trained in Phase 1 and Phase 2 by IHI, UCSF, and TOT trainers, including surgeons, obstetricians, medical officers, midwives, clinical assistants, registered nurses, clinical officers, medical attendants, anesthetists, anesthesiologists, and enrolled nurses.

At the start of Phase 2 meetings were held with IHI project staff, district medical officers, and hospital administrators to review the logistics for implementation, as well as to hear their experiences and challenges during the initial 3 months.

In December 2015, 20 ambulances were equipped with NASGs (one per ambulance) and 21 drivers received training on applying and transporting women with NASGs. As an additional training tool for all levels of facilities, four-minute low resolution (7 MB) and high resolution (11 MB) videos that instructed on NASG application for average, short, large, and unconscious patients were uploaded to the CUG phones during supervision to be used to refresh trained staff or train new staff.

All phases of the training included multiple simulations for a variety of shock and hemorrhage situations (how to transport, placing on conscious and unconscious women, removing when a woman is stable and remains stable, replacing if a woman goes back into shock, etc.). Simulations have been documented to improve retention and team work [37, 38]. We also held unexpected “emergency simulations” during lunch and break times, when participants did not expect to be performing a simulation. An actor playing the husband would carry in an “unconscious and dying,” severely shocked patient/actress and participants had to quickly form a team and demonstrate how they would apply the NASG, manage the hemorrhage and shock of the unconscious and dying actress, and make a referral/transport if necessary.

At the trainings facility staff were informed that they would be receiving the CUG network phones, which became property of the Regional Medical Office. Those who accepted the CUG phones on behalf of the facility signed a contract outlining obligations for maintenance and use, as well as conditions in case of loss or damage. During the Phase 2 training, an introduction to the electronic data collection forms was presented. CEmOC facilities were given onsite training for the electronic forms when their CUG phones were distributed.

Phones distributed for use with the CUG network were Java-enabled Nokia phones. The closed user group was a subscription service through the Airtel company, which provided SIM cards for mobile phones to make calls at no cost to other phones on the network. The CUG phones were also programmed with a data entry system created on OpenXdata. Data was transmitted from the phones via General Packet Radio Service (GPRS) using an http protocol to send data to a local server running a MySQL database for storage and management. The data could be accessed remotely using a secure web-based platform located at: http://maternal.esurveillance.or.tz/.

Entry Criteria/Enrollment: Women were included in the study if they presented with or developed hypovolemic shock secondary to OH at any participating facility. Staff were instructed to apply the NASG for women presenting with signs of obstetric hemorrhage and hypovolemic shock, whether the patient would be referred out or managed on site.

Hemorrhage Management: All facilities had received training on EmOC through the Empower project. Evidence-based guidelines for PPH management included prophylaxis with uterotonics, treatment for PPH with uterotonics, Intravenous fluids, and referral/transport of women who continued to bleed despite uterotonic treatment.

Outcome Indicators: As this was an implementation project, the main outcomes were increased and appropriate use of NASG and CUG network. We set project targets for improvement at 75% increase from the baseline of 0, since both technologies had never been used before. Process indicators included: percentage of facilities with an NASG available and locatable in < 10 min, percentage of health facilities in compliance with established NASG disinfection protocols, percentage of facilities with a CUG network phone available, percentage of facilities able to show a charged CUG network, percentage of facilities that conducted regular training drills, and percentage of phones lost. We also compared baseline case fatality rates (CFR) from OH with endline CFR and predicted a decrease of 25%.

### Data collection

From July –August 2014, IHI staff traveled to each of the 280 sites to collect baseline data. This consisted of reviewing all available hard copy registries to determine the CFR, looking at cases of reported OH and number of deaths attributed to OH for the 12 months from July 1, 2013 – June 30, 2014, the facility-based numbers were compared to the official Regional Reports. Baseline data were also gathered on availability of electricity, presence of cell phones for referrals, and transportation availability for all sites. The number of annual deliveries was gathered from district reporting.

Paper-based data collection on NASG use at the CEmOC facilities occurred from Dec 2014-March 2015, however as not all sites had begun NASG implementation, this data was used for training purposes only and not included in the analysis. CUG network phones were rolled out to all facilities during March–April 2015 during the Phase 2 BEmOC trainings. Both paper forms and electronic data entry forms were written in Swahili.

Facility staff were instructed to complete a case report for each woman with signs of obstetric hemorrhage. This data was recorded on a hard copy register for each site, and later entered into the electronic OpenXdata data entry form on the CUG phone and submitted to the central server by designated health care providers who had been trained for each facility. When there were discrepancies in the electronic records, data was verified against the paper-based register, and if additional verification was required, against other maternity and gynecology registries. In addition, sites sent month-end reports summarizing the number of deliveries, the number of obstetric hemorrhages, and the number of maternal deaths. This data was used to ensure 1) that all facilities were sending data on a regular basis 2) that the total number of OH reported was equal to the number of detailed OH case reports received. In cases of discrepancies or when the month-end report had not been submitted, the project team called facility staff using the CUG network to request the reports.

In addition to the patient data, the data form contained a free text comment field which allowed the provider to supply any additional information. Other fields allowed providers to give the reason they did not use uterotonics, the NASG, or the CUG phone for each patient if applicable.

Besides the quantitative electronic data on patient outcomes, we used a supervision tool to record the process indicators described above to ensure that the NASGs and CUG phones were at the facility and in working condition. These forms were completed by project staff during three rounds of supervision, with all 280 sites visited during each of the three rounds. The final forms were completed during the April–May 2016 supervision as an endline measure.

On-site supervision was conducted three times throughout the 15 months: from July – August 2015, December 2015 – January 2016, April – May 2016. Each site received three visits by project staff, at which time they were asked to demonstrate their skills, electronic reports were verified against paper records, and troubleshooting was performed for the phones. The physical condition of the NASGs and phones were verified and recorded on the supervision forms along with the number of staff trained, the number of staff not yet trained, and the frequency of drills.

### Qualitative data collection

Feedback was sought to further enhance training and use of CUG and NASG. In addition to the patient data, the data forms contained the free text comment field noted above, which allowed the provider to supply any additional information. Qualitative informal interviews were conducted with facility staff during all three rounds of routine supervisory visits for sites, focusing on sites with apparent overuse (use on women not meeting criteria) or with lower numbers of uses than predicted.

For 2 weeks in July 2015, formal interviews were conducted with 19 facility staff, 15 of them working at the dispensary level, about their experiences with NASG. Thirteen of the staff had been trained, but not yet had a patient that required application of the NASG. Questions focused on ease of use, reasons for overuse or non-use, suggestions for improving training, and NASG acceptability. Due to the timing during the earlier stage of the project, most of the interviews focused on providers’ confidence and theoretical concerns following their training, as few providers had prior experience.

Data analysis Quantitative data analysis was conducted using R (R Core Team (2017). R: A language and environment for statistical computing. R Foundation for Statistical Computing, Vienna, Austria. URL https://www.R-project.org/) and Stata 12.1 (StataCorp. 2011. *Stata Statistical Software: Release 12*. College Station, TX: StataCorp LP). Cohort study Risk Ratio (RR) methods within Stata were used to compute relative risk and Chi-Square statistics for the association of the use of the NASG with case fatality rates. Qualitative data analysis was conducted by seeking themes within the various responses to open-ended fields and to the structured one to one interviews.

## Results

One thousand seven-hundred thirteen women with obstetric hemorrhage from any etiology were observed during the 15-month data collection period, with reporting from 276 facilities (98.6%). Estimated median blood loss was 500 mL (*n* = 1672, 97.6%), range 0 ml–3500 ml). Only 5.4% (*n* = 92) of women were missing blood pressure recordings and 5.6% (*n* = 96) were missing pulse, but consciousness level was recorded for all women. Blood loss estimates were missing for 3.0% (*n* = 51) of women. Additionally, 1.4% of facilities (4 dispensaries) failed to send any reports (monthly totals or individual cases).

**Fig. 2:**
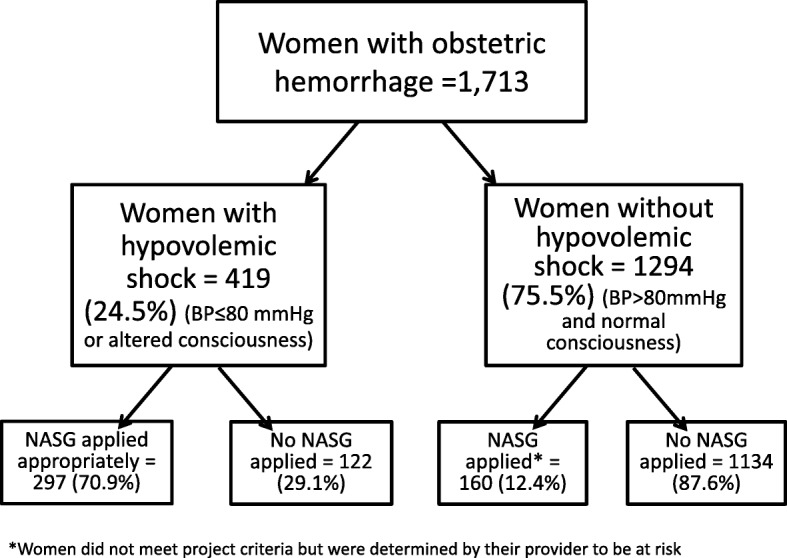
Use of NASG for Women with Hypovolemic Shock Secondary to Obstetic Hemorrhage from April 1, 2015-June 30, 2016

Table 1 compares conditions between women who had OH (*n* = 1713), but did not receive the NASG (*n* = 1256), and those women who had OH and did receive the NASG (*n* = 457). The majority of the women who received the NASG had lower SBPs and were more likely to have altered level of consciousness. Of the 1713 women with OH, 419 (24.5%) met project hypovolemic shock criteria. Table 2 compares only those women who met study criteria of hypovolemic shock secondary to OH (*n* = 419), and shows worsening conditions and rates of use for different etiologies among those who received the NASG (*n* = 297) and women who did not receive the NASG (*n* = 122). As shown in Fig. 2, the NASG was applied to 70.8% (*n* = 297) of these women. Of the subgroup of 225 women (53.7%) experiencing shock who were transported to higher level care, 85.8% (*n* = 193) had the NASG applied before transfer, exceeding project targets of 75%. Of these 225 women transferred, 164 (72.8%) had referral communication using the CUG network.

**Table 1:** Characteristics of all women with Obstetric Hemorrhage (*n* = 1713), those who received the Non-pneumatic Anti-Shock Garment (*n* = 297), and those who did not receive the Non-pneumatic Anti-Shock Garment (*n* = 122)

Variables	Women with NASG applied n (%)	Women without NASG applied n (%)	Total Frequency (*n* = 1713)
Systolic blood pressure			
≤ 80	183 (73.2)	67 (26.8)	250 (100)
81–90	113 (47.7)	124 (52.3)	237 (100)
> 90	147 (13.0)	987 (87.0)	1134 (100)
Missing	14 (15.2)	78 (84.8)	92 (100)
Consciousness level			
Normal	230 (16.4)	1176 (83.6)	1406 (100)
Confused/unconscious	227 (73.9)	80 (26.1)	307 (100)

**Table 2:** Characteristics of women who met study criteria of hypovolemic shock secondary to obstetric hemorrhage (*n* = 419) who received non-pneumatic anti-shock garment (*n* = 297) and women with hypovolemic shock who met criteria, but did not receive non-pneumatic anti-shock garment (*n* = 122)

Variables	Women with NASG applied *n* (%)	Women without NASG use *n* (%)	Total frequency *n* = 419 *n* (%)
Referred to higher care			
Yes	193 (85.8)	32 (14.2)	225 (100)
No, managed at facility	104 (53.6)	90 (46.4)	194 (100)
NASG applied			
Yes	297 (100)	0 (0)	297 (71)
No	0 (0)	122 (100)	122 (29)
Etiology			
Complications of abortion	46 (41.8)	64 (58.2)	110 (100)
Uterine atony	65 (84.4)	12 (15.6)	77 (100)
Retained placenta	50 (79.4)	13 (20.6)	63 (100)
Placenta previa	33 (78.6)	9 (21.4)	42 (100)
Placental abruption	30 (85.7)	5 (14.3)	35 (100)
Ruptured uterus	25 (73.5)	9 (26.5)	34 (100)
Lacerations	23 (88.5)	3 (11.5)	26 (100)
Ectopic pregnancy	4 (66.7)	2 (33.3)	6 (100)
DIC	5 (83.3)	1 (16.7)	6 (100)
Other	16 (80.0)	4 (20.0)	20 (100)
Systolic blood pressure			
≤ 80	183 (73.2)	67 (26.8)	250 (100)
81–90	52 (76.5)	16 (23.5)	68 (100)
> 90	56 (59.6)	38 (40.4)	94 (100)
Missing	6 (85.7)	1 (14.3)	7 (100)
Consciousness status			
Normal	70 (62.5)	42 (37.5)	112 (100)
Confused	148 (75.5)	48 (24.5)	196 (100)
Unconscious	79 (71.2)	32 (28.8)	111 (100)

A comparison of CFR at baseline (1.70) to CFR at the end of the project (0.76) showed a 67% reduced risk for women during the project ([RR: 0.33, 95% CI = .19, .60], not shown).

Supplemental qualitative data was voluntarily added by providers for 66.4% (*n* = 1138) of the hemorrhage cases in the free text comment field of the data screen: This field was frequently used to send positive feedback regarding the program such as:

“NASG helped, patient came to normal 120/60.” (Was 70/40).

“NASG has helped the patient keep well”.

“Died and back, NASG saves lives.”

*Nasg inaokoa maisha ya akina mama* “NASG saves mother’s lives.”

*Mgonjwa alishukuru sana watoa huduma vazi lilimuokoa “*The patient thanked the service providers who had saved her”.

Challenges were also reported, mainly when ambulances or other transport were called, but did not arrive for referrals, resolving missing data on hard copy records with electronic records, and additional support requests for training or referrals.

Interview data theme analysis showed the following categories of obstacles and opportunities in the early days of the implementation project: perceived training needs, limitations to cascade training, desire for experience, and transportation difficulties [39]. From these early interviews, we learned how to improve the training and implementation of the project. Due to low patient volume at the dispensaries, these providers expressed fears that they anticipated not being proficient enough to apply the NASG correctly. There was a belief expressed that perhaps practice should occur on live patients in a hospital environment, rather than in a controlled simulation of the training center.

### Process indicators

Data collected during endline supervision are reported in Table 3. At endline, 280 facilities (100%) had an NASG and 271 (96.8%) had all their NASGs disinfected, laundered, folded, and stored in an area where they were not exposed to potential damage by pests. The only indicator where facilities did not exceed targeted outcomes, was that only 43% reported conducting regular training drills.

**Table 3:** Endline supervision of facility process indicators *N* = 280 FACILITIES

Indicator	Baseline	Endline	Target
Percentage of facilities that have a NASG available and can locate it in less than 10 min (*n* = 280)	0 0%	280 100%	210 75%
Percentage of higher level health facilities in compliance with established NASG disinfection protocols (*n* = 280)	0 0%	272 97.1%	210 75%
Percentage of facilities able to produce a charged functional CUG network mobile phone within 15 min	0 0%	274 97.9%	210 75%
Percentage of facilities that conduct regular training /drills on NASG and CUG protocols (*n* = 280)	0 0%	120 42.8%	210 75%
Percentage of phones lost (*n* = 590)	0 0%	35 6%	59 10%

## Discussion

This large scale implementation project in rural Tanzania demonstrated that appropriate utilization rates of 71% of women in hypovolemic shock could be achieved, even in lower level health care facilities. Positive contributing factors included adequate, frequent training and supportive supervision to use the NASG, clear definitions and parameters of hypovolemic shock, rapid and responsible access from project management and clinicians via the CUG, and intensive follow up for every death that occurred and for cases of non-use with women who met eligibility criteria. Although there was some use on women who did not meet the hypovolemic shock criteria, none of these women suffered side effects or morbidities/mortalities. The study also showed a temporal association of a 67% decrease in CFR in the study facilities at the project endline.

Alongside the near universal use of NASG for critically ill women with obstetric hemorrhage reported from one high-level facility in Cali, Colombia [27], this project demonstrated high utility among providers in very different settings and among different types of providers in rural Tanzania. Likewise, in Tamil Nadu State in India, the NASG has almost universal application at all levels of health care facilities, including on all 108 Emergency Management and Research Institute ambulances throughout the state [40]. In Timor Leste among ambulances and lower level facilities, there was uptake of NASG, including use on 40/86 PPH cases in only a 10-month period. The Colombia, Tamil Nadu, Timor Leste results, and this report contradict a finding of low utilisation from an evaluation of a national implementation project in Niger, in which misoprostol, UBT, and NASG were introduced. Of the 59 maternal deaths reviewed, only 4 women received the NASG [33].

However, the protocol for that project instructed that the NASG should not be applied for every woman with PPH. Rather, a sequenced approach was used, patients were given prophylactic misoprostol, then, if a hemorrhage occurred, the woman was treated with misoprostol. If the woman continued bleeding, a UBT was inserted. If the above treatments did not stop the bleeding, then NASG would be applied [26]. The low percentage of uses reported might indicate that the first two steps (miso and UBT) avoided some of the need for the NASG. However, limitations around primary data collection make it difficult to determine. In conversation with a national trainer from Health and Development International (HDI), the implementing organization working with the MOH in Niger (Zeidou A 2018, oral communication 14 August 2018), we learned that the Niger project did not keep its own data throughout the project; they attempted to do so in 2015, but following low (approximately 5%) reporting they were asked by government partners to halt and transition to the use of official government regional reporting. Beginning in 2016 they only utilized MOH reporting. For these reasons, the evaluation team conducted its own data collection, by reviewing the case records on only 59 (35%) of the 171 reported deaths. Finding a use of NASG on only 7% of the women included in the mortality review, may not reflect low use of NASG. It might, instead, be more interesting to see the numbers/percentages of use on women with severe hemorrhage/hypovolemic shock who did not die. Since that data is not available, it seems improbable that this low rate of NASG can be generalizable to the other women in this project or to any other project. While we agree that hypovolemic shock secondary to OH is a rare event, it is still possible to demonstrate adequate use with more reliable data.

It is also possible that differentials between other reports and the Niger report could be due to different approaches to training in the Empower project (frequent doses, higher use of simulations and practice), supportive supervision, close follow-up of cases, or use of primary concurrently collected data as seen in Tanzania and Timor Leste. Lower utilization rates, < 35% of PPH cases, found in two Nigerian states, Ibadan [30] and Ondo [28], may also reflect these differences in training, supply chain, exchange and return plans, political support, policy support, regulations over who can use NASGs (not allowing midwives, the front line workers, to use NASGs) [30]. They might also reflect examining rates of use against all OH cases, rather than only the severe cases with hypovolemic shock.

However, even the projects reporting lower than expected use can impart important lessons about project implementation of new technologies. We note in our study and in others a preference for onsite training with real OH cases, more hands-on skills sessions, and that the rarity of severe PPH may result in loss of skills’ competencies. We noted this in our first round of qualitative interviews [39]. The Tanzania project seemed to have reduced some of these problems, first in the initial planning and then through course corrections responding to feedback; instituting a phased approach with adequate time during training for hands-on skills practice and team work; time to learn lessons and make course corrections before widespread implementation; continuous real-time review of hemorrhage case reports to determine whether the NASG was being used appropriately on women with hypovolemic shock, with follow-up conducted by CUG for facilities/individual staff that had problems with compliance in use of the NASG or reporting data; ability to contact project staff over the CUG system when challenges or questions arose; ability of district staff who were certified during the TOT to provide supervision and retraining during their routine quarterly site visits; encouragement of skills practicum sessions at the facility level to overcome rarity of severe PPH cases; and the ability for facility staff to view training videos on the CUG phones.

In Tanzania the utilization by already trained providers of a new emergency obstetric first-aid device for severe hemorrhage cases, which often cause high anxiety and fear, involves a difficult behavior change [41–43]. The Empower providers have been dealing with severe hemorrhage and maternal mortality, often for years, and often with horrible outcomes. Intensive hands-on simulation training during the project may have helped overcome this obstacle. Having the videos on their phones may have also decreased anxiety as newly trained health care workers had a readily available model to show application and management. These providers were already highly motivated by participation in Empower I and II, already updated on PPH management, EMOC, and improving referrals.

In some reports which recommend more hands-on training, they also suggest that the training might be costly. In this project, except for the two original TOTs, training for NASG/CUG was incorporated into ongoing EmOC training. Another question about costs, involve the CUG system and its sustainability. While we did not do a formal cost analysis, Regional Tanzanian government officials managing health services already had a budget equal to $175 USD per month to cover communications costs for four officials in a single region. This is equal to the monthly cost for the entire CUG network which covered all the health facilities in one district, as well as the District Medical Officers, who also received phones as part of the project. There are also comments about the “high costs” of the NASG. For this project, the initial purchase price was $55.00/per NASG. As the NASG can be washed and re-used multiple times, this results in a highly cost-effective management tool [19, 44, 45].

### Limitations

While not a limitation to an implementation project, limitations to this project’s generalizability should be noted. This was not a randomized clinical trial, we compared endline process indicators results to baseline rates of 0 at baseline where there were no NASGs or CUGs available. We did encounter some difficulties with data collection on paper forms and with referrals on the providers’ own cell phones, this resulted in the switch to the CUG phone network and electronic data collection. The temporal differences since baseline, such as participation in Empower trainings could also have improved the providers’ EmOC skills; there may have been an increase in the number of women who received appropriate prophylaxis and/or treatment uterotonics during the intervention period as compared to the baseline period. Included in limitations were limitations to the training, particularly during the first few months, however, we did collect qualitative data on obstacles, and were able to respond and make course corrections in the training to emphasize even more simulations, practicums, and hands-on experiences. Total reporting was from 98.6% of all facilities, with all non-reporting facilities being dispensaries. Reports were received from all referral sites. The facilities that were unable to send reports had low network coverage or other technical obstacles, including being too close to the Zambian border to connect to a Tanzanian network. We attempted to overcome these difficulties by retrieving hard copy forms, sending a local project technician to troubleshoot the phones, or by having facility staff travel to an area with service in order to send monthly reports; however 4 facilities did not succeed in sending reports and were excluded from analysis. The emphasis was placed on ensuring all the referral facilities had complete reporting. There are methodological limitations with comparing CFR at baseline to CFR at endline, including the fact that other factors besides the NASG that could have affected the CFR outcomes during the project period. We will conduct further analyses on this data to investigate this association.

### Strengths

However, there are many strengths to this project. First this was a real-world implementation project in rural facilities with a high need for overcoming delays in distance and in lack of definitive shock treatment at lower levels. There were high numbers of facilities (280) across a number of levels of a health care system. There were high response rates among facilities participating in the project, in the trainings, and in reporting. Embedding the NASG/CUG components into overall EmOC training was a strength, and also improved cost-effectiveness vs. holding a separate, stand-alone training. Management referrals and follow up with the CUG was a big strength, which contributed not only to the high response rates and validation of statistics, but also to case referral follow up. Evidence-based training in management of OH in the Empower II Project, followed, in our trainings, with clear objective clinical parameters of hypovolemic shock secondary to OH (not just an unreliable blood loss estimation), enabled the providers to appropriately apply NASGs to those women who really needed them. Another method of ascertaining accuracy in utilization was the careful follow-up of mortalities and non-use through continuous review of incoming OH reports and month-end reporting accompanied with follow-up by CUG phone if reports were not received, if the facilities reported fewer than anticipated OH cases, or if case management appeared inconsistent with protocols for appropriate NASG use. If phone calls did not work to improve both reporting or use, the issue was escalated to facility management and ultimately district level management for in-person follow-up by a senior trainer. While these are not necessary in all implementation studies, this rigor did allow the project to demonstrate that high utilization is possible. At the end of the project, the region of Singida had generated sufficient government support to enable the region to independently scale up to the three districts that had not been included in the project, using their regional trainers and purchasing the necessary NASGs. For sustainability, the intensive follow up and data collection could be eliminated, but the simulation training and emphasis on supportive supervision could be more affordably sustained.

## Conclusion

Despite few existing reports on real world use of the NASG within rural health care settings, this study of 276 reporting facilities and over 1000 providers supported via CUG demonstrated that NASG can have high uptake with appropriate use for hypovolemic shock secondary to OH. A phased introduction, practical hands-on training, supportive supervision as needed through the CUG network, real-time monitoring of OH case reports, and ability to watch training videos on the phones seem to be keys to acceptability and utilization. Incorporating NASG/CUG training into already established EmOC training and implementations also enhanced program success. It is clear by comparison to projects with fewer or lower quality and/or stand-alone training, supervision, communication, and referrals that a one-time training followed by distribution of the NASG to clinics and facilities might result in low utilization. Adding NASG training to pre-service curricula may further enhance uptake for new nurses, midwives, doctors, and other skilled maternal health care workers, who will arrive at their new posts already possessing skills and confidence in NASG application, management, and removal. With the proper implementation strategies, NASG utilization can be high and should be associated with decreased mortality among mothers at risk of death from obstetric hemorrhage.
